# Poster Session II - A297 CASE REPORT: SINGLE DOSE OF INFLIXIMAB AND RISK OF ANTIBODIES

**DOI:** 10.1093/jcag/gwaf042.297

**Published:** 2026-02-13

**Authors:** C Pray, C Galts, A Elgaml, M Elsayyad, K J Khan

**Affiliations:** Gastroenterology, McMaster University, Hamilton, ON, Canada; Gastroenterology, McMaster University, Hamilton, ON, Canada; McMaster University, Hamilton, ON, Canada; McMaster University, Hamilton, ON, Canada; McMaster University, Hamilton, ON, Canada

## Abstract

**Background:**

Antibody testing pre-infliximab re-initiation is not available in Canada.

**Aims:**

Review an aggressive Crohn’s case

**Methods:**

A 32 year-old previously healthy male was admitted after a few days rectal bleeding and diarrhea with CT showing features of acute pancolitis presumed to be infectious. He was treated conservatively with negative stool cultures and a sigmoidoscopy demonstrating acute inflammation and biopsies not suggestive of IBD. After 1 week, he did not improve and had full colonoscopy (terminated at transverse colon) with severe colitis seen now presumed to be IBD.

**Results:**

He responded to IV steroids and switched to oral after 3 days. After 2 days of oral steroids, he had a recurrence of symptoms and infliximab was started. One day after starting infliximab (one dose given), he had massive lower GI bleed with hemodynamic instability. CT confirmed a transverse colon arterial bleed that was unsuccessfully embolized leading to emergency subtotal colectomy with end-ileostomy. Infliximab was not continued and 4 months later, ileoscopy and sigmoidoscopy were normal. Six months after re-anastamosis, sigmoidoscopy showed 3 small ulcers at anastomosis (Rutgeerts i2) but normal ileum and distal colon. Three months later he presented with new rectal bleeding and repeat sigmoidoscopy showed multiple ulcers (Rutgeerts i3) and ulcers in neo-ileum. The patient elected to start upadacitinib. Nine months later he had improved symptoms but formed a new fistula in ano requiring seton placement. He also had notable acne and it was decided to re-initiate infliximab again. It was presumed safe to restart infliximab with low risk of antibody to infliximab (ATI). He was not started on immunomodulator but had hydrocortisone pre-infusion. He had a favourable clinical response to infliximab, and at 6 months, we repeated his sigmoidoscopy which showed almost normal mucosa with small ulcer seen (Rutgeerts i1) but his infliximab levels were undetectable and ATI was markedly elevated at > 875 AU/mL. We decided to change his therapy again.

**Conclusions:**

Infliximab re-induction after drug holiday should be preceded by ATI testing which is currently not available in Canada. In this case, robust ATI formation occurred despite a good clinical response and only 1 infliximab dose prior. Concomitant hydrocortisone was administered, but immunomodulator could also be considered in future cases to mitigate antibody risk. The rate at which ATI’s become undetectable following treatment cessation remains uncertain; however, it is postulated that antibodies may be undetectable within 12 months of therapy cessation. Accordingly, it would be reasonable to offer ATI testing when re-inducing therapy within 12 months of a drug holiday, though additional data are needed to better define this timeframe and its clinical implications.

**Funding Agencies:**

None

